# Editorial: Direct and Indirect Interactions of HIV With Host Cells

**DOI:** 10.3389/fcimb.2021.771370

**Published:** 2021-10-11

**Authors:** Tetsuo Tsukamoto, Santhi Gorantla, Vasco Rodrigues

**Affiliations:** ^1^ Department of Immunology, Faculty of Medicine, Kindai University, Osaka, Japan; ^2^ Department of Pharmacology and Experimental Neuroscience, College of Medicine, University of Nebraska Medical Center, Omaha, NE, United States; ^3^ Institut Curie, PSL Research University, INSERM U932, Paris, France

**Keywords:** human immunodeficiency virus (HIV), acquired immunodeficiency syndrome (AIDS), virus-host interaction, hematopoietic stem/progenitor cells (HSPCs), NeuroHIV

HIV causes AIDS by infection and depletion of CD4^+^ T cells. HIV can also infect other CD4^+^ cells such as macrophages, possibly leading to their functional alterations and contribution to viral reservoirs (Hendricks et al., 2021). HIV infection may also result in changes in other hematopoietic cells such as hematopoietic stem/progenitor cells (HSPCs), CD8^+^ T cells, B cells, natural killer cells, dendritic cells (DCs), and granulocytes (Virgilio and Collins, 2020; Dillon and Wilson, 2021; Madzime et al., 2021). Furthermore, HIV infection can even cause alteration of non-hematopoietic cells. Cells in various tissues and organs, including bone marrow, thymus, gut, genital tract, lung, and central nervous system, have been investigated in depths in HIV-infected hosts (Dillon and Wilson, 2021; Pruitt, 2021). However, the outstanding breadth of the direct/indirect influence of HIV on host cells in different tissues and organs could make it challenging to grasp the whole picture of the HIV disease.

In three articles on this Research Topic, scientists investigated the impact of HIV-1 on host immune cells. Perot et al. extended their previous study on tetraspanin 7 (TSPAN7)-dependent transfer of HIV-1 from immature monocyte-derived DCs (iMDDCs) to CD4^+^ T cells (Ménager and Littman, 2016). They discovered that TSPAN7 expression is downregulated in the process of DC maturation and that viral replication-independent TSPAN7-dependent trans-infection of HIV-1 in iMDDCs is far more efficient than the replication-dependent, TSPAN7-independent virus transfer mechanism in mature monocyte-derived dendritic cells, further clarifying the previously proposed two-phase trans-infection model. Bharucha et al. analyzed the impact of β-defensins hBD2 and 3 on HIV-1. Defensins are small peptides with potent antimicrobial effects (Brice and Diamond, 2020). The defensins hBD2 and 3 were previously shown to inhibit HIV replication in CD4^+^ T cells. In this article, the authors demonstrated for the first time that the two defensins also inhibit HIV replication in monocyte-derived macrophages (MDMs). Intriguingly, they described the potential effect of hBD2 treatment on the upregulation of antiviral factors APOBEC3A and APOBEC3G. Further study will clarify whether β-defensins can be used as therapeutics and whether they influence viral reservoirs in MDMs. Badura et al. investigated B-cell defects observed in HIV-1-infected patients. The authors detected altered B-cell dynamics during acute HIV-1 infection caused by impaired bone marrow output of naïve B cells, which correlated with the degree of T-cell activation, and increased B-cell proliferation. The impaired bone marrow output was more profound in patients with severe disease, suggesting the importance of correcting bone marrow abnormalities in HIV-1 infection. Furthermore, the impact of 8-week antiretroviral therapy (ART) on bone marrow B-cell output was limited.

The topic also highlights HSPCs in HIV infection. In his mini-review, Tsukamoto summarizes recent advances in our understanding of the interaction between HIV and HSPCs. He describes the current significant issues on viral reservoirs and the impact of HIV on the number, function, and aging of HSPCs. Bordoni et al. provide evidence on the relationship between chronic HIV infection and aging in circulating CD34^+^ hematopoietic progenitor cells (HPCs). In the study, peripheral HPCs from HIV^+^ patients with low CD4^+^ T-cell counts (<500/mL blood) showed decreased *in vitro* white colony-forming cell (CFC) capacities. The authors further indicated that increased expression of Period circadian clock 2 (Per2) in HIV^+^ patients might have caused lower white CFC counts. Furthermore, they demonstrated the association of HIV infection with the downregulation of Sirtuin 1 (Sirt1), a histone deacetylase and potential anti-aging protein. The Sirt1 inhibitor resveratrol restored the Sirt1 expression levels and caused downregulation of Per2 *in vitro*. These exciting results stress the need to compare HPCs between healthy and HIV^+^ individuals using a broader panel of host factors affecting cellular processes such as senescence and inflammatory response. Valverde-Villegas et al. provide data showing the distinct distribution of cells with HSPC-like phenotypes in breast milk and peripheral blood between HIV^+^ and HIV^−^ young women. According to the data, CD45^+^CD34^−^CD133^+^ cells showed increased frequencies in milk and blood of HIV^+^ women. However, the increase in the frequency of the CD133^+^ cells was not associated with proinflammatory cytokines in milk except IL-8. The intriguing results will stimulate further research on the origin and the fate of these primitive stem/progenitor-like cells.

The topic further includes three reviews on the central nervous system (CNS). Gorska and Eugenin describe the dysregulation of glutamate in HIV-associated neurocognitive disorders (HAND). The authors detail how overproduction of glutamate, the critical neurotransmitter, can induce cytotoxicity and chronic inflammation. In the CNS, ART cannot block the production of viral proteins gp120, Tat, and Vpr in HIV-infected microglia and astrocytes, which may be associated with inflammatory responses. In addition, persistent activation of glial cells causes increased synthesis and release of glutamate, leading to overstimulation of glutamate receptors and neurotoxicity. Ajasin and Eugenin review previous articles facilitating the current understanding of Tat in the CNS and cardiovascular diseases. The authors detail the role of Tat not only in HIV-infected cells but also in uninfected bystander cells such as microglia and astrocytes. Following the uptake of Tat released from HIV-infected cells, bystander cells produce neurotoxic factors, including proinflammatory cytokines. Lastly, Tice et al. feature the glymphatic system, i.e., a system for waste clearance in the CNS, and the water channel aquaporin 4 expressed on astrocytic endfeet. There has been limited evidence to show changes in AQP4 in HIV infection. However, given the pathological similarities between NeuroHIV and Alzheimer’s disease that accompanies altered distribution and expression of AQP4 (Yang et al., 2016) and previous observations with macaque AIDS models, the authors propose that it is time to investigate the potential impact of viral latency/reactivation in the brain on disruption of the glymphatic system.

In summary, our Research Topic brings together nine articles that describe novel findings, up-to-date information, or future directions to advance our understanding and thinking of host cells in connection with HIV infection. Although the funding environment surrounding basic HIV research has become increasingly complex (Levy, 2020), we hope that this Research Topic will help encourage researchers who work on the elucidation of HIV pathogenesis for better prevention and treatment in the future.

## Author Contributions

TT, SG, and VR edited the Research Topic, wrote, and approved the manuscript.

## Funding

TT has been supported by JSPS KAKENHI (grant numbers JP19K07581 and JP19H03481) and Mochida Memorial Foundation for Medical and Pharmaceutical Research.

## Conflict of Interest

The authors declare that the research was conducted in the absence of any commercial or financial relationships that could be construed as a potential conflict of interest.

## Publisher’s Note

All claims expressed in this article are solely those of the authors and do not necessarily represent those of their affiliated organizations, or those of the publisher, the editors and the reviewers. Any product that may be evaluated in this article, or claim that may be made by its manufacturer, is not guaranteed or endorsed by the publisher.
